# Next-generation metabolic screening: targeted and untargeted metabolomics for the diagnosis of inborn errors of metabolism in individual patients

**DOI:** 10.1007/s10545-017-0131-6

**Published:** 2018-02-16

**Authors:** Karlien L. M. Coene, Leo A. J. Kluijtmans, Ed van der Heeft, Udo F. H. Engelke, Siebolt de Boer, Brechtje Hoegen, Hanneke J. T. Kwast, Maartje van de Vorst, Marleen C. D. G. Huigen, Irene M. L. W. Keularts, Michiel F. Schreuder, Clara D. M. van Karnebeek, Saskia B. Wortmann, Maaike C. de Vries, Mirian C. H. Janssen, Christian Gilissen, Jasper Engel, Ron A. Wevers

**Affiliations:** 1 Department of Laboratory Medicine, Translational Metabolic Laboratory (TML), Radboud University Medical Center, Geert Groote Plein Zuid 10, 6525 GA Nijmegen, The Netherlands; 2 Department of Human Genetics, Donders Institute of Neuroscience, Radboud University Medical Center, Nijmegen, The Netherlands; 3 Department of Clinical Genetics, Laboratory of Biochemical Genetics, Maastricht University Medical Center, Maastricht, The Netherlands; 4 Department of Pediatric Nephrology, Amalia Children’s Hospital, Radboud University Medical Center, Nijmegen, The Netherlands; 5 Department of Genetic Metabolic Disorders, Emma Children’s Hospital, Academic Medical Center, Amsterdam, The Netherlands; 6 Department of Pediatrics, Salzburger Landeskliniken (SALK) and Paracelsus Medical University (PMU), Salzburg, Austria; 7 Department of Pediatrics, Amalia Children’s Hospital, Radboud University Medical Center, Nijmegen, The Netherlands; 8 Department of Internal Medicine, Radboud University Medical Center, Nijmegen, The Netherlands; 9 Institute for Molecules and Materials, Radboud University Nijmegen, Nijmegen, The Netherlands

**Keywords:** Metabolomics, Biomarkers, High-resolution, QTOF, Mass spectrometry, Innovative laboratory diagnostics, Inborn errors of metabolism, Canavan disease, Xanthinuria

## Abstract

The implementation of whole-exome sequencing in clinical diagnostics has generated a need for functional evaluation of genetic variants. In the field of inborn errors of metabolism (IEM), a diverse spectrum of targeted biochemical assays is employed to analyze a limited amount of metabolites. We now present a single-platform, high-resolution liquid chromatography quadrupole time of flight (LC-QTOF) method that can be applied for holistic metabolic profiling in plasma of individual IEM-suspected patients. This method, which we termed “next-generation metabolic screening” (NGMS), can detect >10,000 features in each sample. In the NGMS workflow, features identified in patient and control samples are aligned using the “various forms of chromatography mass spectrometry (XCMS)” software package. Subsequently, all features are annotated using the Human Metabolome Database, and statistical testing is performed to identify significantly perturbed metabolite concentrations in a patient sample compared with controls. We propose three main modalities to analyze complex, untargeted metabolomics data. First, a targeted evaluation can be done based on identified genetic variants of uncertain significance in metabolic pathways. Second, we developed a panel of IEM-related metabolites to filter untargeted metabolomics data. Based on this IEM-panel approach, we provided the correct diagnosis for 42 of 46 IEMs. As a last modality, metabolomics data can be analyzed in an untargeted setting, which we term “open the metabolome” analysis. This approach identifies potential novel biomarkers in known IEMs and leads to identification of biomarkers for as yet unknown IEMs. We are convinced that NGMS is the way forward in laboratory diagnostics of IEMs.

**Electronic supplementary material:**

The online version of this article (10.1007/s10545-017-0131-6) contains supplementary material, which is available to authorized users.

## Introduction

The number of known inborn errors of metabolism (IEMs) has grown substantially in recent decades, amounting to ~1000 individual conditions, with accumulative incidence of ~1:1000 newborns. For a selection of treatable IEMs, routine newborn screening (NBS) has been implemented for early diagnosis. However, most IEMs are not covered in NBS, and for those conditions, diagnostic profiling in the metabolic laboratory is indispensable to reach a correct diagnosis for an individual patient. The current diagnostic toolbox for IEM screening comprises a panel of targeted analyses based on a variety of analytical techniques, including gas chromatography mass spectroscopy (GC-MS), liquid chromatography tandem mass spectroscopy (LC-MS-MS), and ion-exchange chromatography. The metabolic laboratory therefore must maintain a substantial number of different analyzers and use laborious manual methods. Due to time and logistic restraints, it is not feasible to apply all possible methods for IEM screening to each individual patient. Therefore, in current practice, clinical symptomatology is leading in the selection of specific analyses for an individual patient. However, this strategy heavily relies on the completeness of clinical information provided to the metabolic laboratory and therefore holds the risk of false-negatives if a metabolic test has not been performed due to incomplete description of patient symptoms. Additionally, the discovery of novel metabolic defects is likely hampered by investigating only a limited selection of known metabolic pathways. Thus, there is a demand for a holistic approach to metabolite analysis in IEM screening, and technologies to fulfill this unmet clinical need are emerging: advanced, high-resolution mass spectrometry (MS) enable untargeted investigation of the metabolite profile, i.e. a holistic overview of small molecules with a mass <1500 Da in a biological system at a certain time point. In analogy to other holistic “–omics” technologies, it is referred to as “metabolomics” (Nicholson et al. 1999). Untargeted metabolomics is widely applied to generate fundamental mechanistic insights in research fields ranging from environmental science, to toxicology, to human diseases in which cancer, cardiovascular disease, and diabetes have been main subjects (Johnson et al. 2016). In IEMs, untargeted metabolomics is gradually emerging (Tebani et al. 2016). In the 1990s, application of high-resolution proton nuclear magnetic resonance (NMR) for diagnosing IEMs was developed in our laboratory, which led to the identification of several new IEMs (Iles et al. 1995; Wevers et al. 1995). However, NMR spectroscopy did not evolve as a common IEM screening technique, likely because of the financial constraints and relatively low sensitivity (Emwas et al. 2013). High-resolution liquid chromatography (LC)-MS-based metabolomics can overcome this sensitivity problem, as the detection limit is in the low nanomolar range, compared with the low micromolar range of NMR. In 2007, Wikoff and co-workers evaluated the potential of untargeted LC-time-of-flight (TOF) MS for classical methylmalonic aciduria (MMA (*mut*^0^), OMIM #251000) and propionic aciduria (PA, OMIM #606054). Their proof-of-concept study identified known biomarkers and showed that an untargeted approach increases the possibility of identifying new biomarkers in known disorders (Wikoff et al. 2007). A similar untargeted metabolomics study on patients and obligate heterozygotes for isovaleryl-CoA dehydrogenase deficiency (IVA, OMIM #243500) demonstrated a clear metabolic discrimination between these groups and identified different metabolic profiles in treated and untreated IVA patients (Dercksen et al. 2013). Also, untargeted LC-MS metabolomics in urine distinguished types I and II xanthinuria profiles (Peretz et al. 2012). Another example was the promising evaluation of an untargeted high-resolution MS method for analysis of dried blood spot (DBS) samples for NBS on phenylketonuria (PKU, OMIM #261600) and medium-chain acyl-CoA dehydrogenase deficiency (MCADD, OMIM #201450) (Denes et al. 2012). Additionally, several applications of untargeted metabolomics to other IEMs have been published, including respiratory-chain defects, mucopolysaccharidosis type I, and infantile cerebellar–retinal degeneration associated with mitochondrial aconitase (ACO2) deficiency (Smuts et al. 2013; Venter et al. 2015; Tebani et al. 2017; Abela et al. 2017).

In all IEM metabolomics studies referred to above, untargeted metabolomics was applied to predefined patient groups, and statistical analysis was performed by comparing control versus patient groups to identify disease-specific biomarkers. However, the application of high-resolution metabolomics in routine diagnostic screening for IEMs requires methodology that can robustly profile individual patients without prior knowledge of the diagnosis. In this journal, Miller et al. previously reported an untargeted metabolomics approach for screening individual patients for IEMs (Miller et al. 2015). Even though that study showed promising results, it involved a dual-platform approach, and resolution was not optimal, as the amount of individual metabolites identified was not on a “big-data” level, and key IEM biomarkers were missed (e.g., guanidinoacetate, methylmalonate, tetradecenoylcarnitine, and tetradecadienoylcarnitine). High-resolution metabolite detection is indispensable to fully exploit the possibilities of metabolomic approaches for both diagnosing known IEMs and identifying novel diseases and/or biomarkers in individual patients.

We present a single-platform, untargeted, high-resolution LC quadrupole time-of-flight (QTOF) metabolic profiling method that can be applied in the diagnostic screening for IEMs in individual patients, which we named next-generation metabolic screening (NGMS). We clinically validated the diagnostic performance of our NGMS strategy through analysis of plasma samples from patients with 46 distinct IEMs. Using our analytical and semiautomated data-processing approach, we detected >10,000 features—i.e., signals with a specific mass-to-charge ratio, intensity, and retention time—in a plasma sample of an individual patient. To extract clinically relevant metabolite/feature information on IEM diagnosis, we selected features that significantly different between individual patients and controls and cross-referenced them to a panel of 340 known IEM-related metabolites. As a subsequent step, the full metabolomic profile is available for exploratory untargeted analysis. In this study, we focus on results of the clinical validation study and show examples of the added clinical value of NGMS-based diagnostics in IEMs.

## Methods

### Sample collection

For a spectrum of 46 distinct IEMs (Table 1), plasma samples were available. Heparin blood samples of these patients was drawn for routine metabolic screening or follow-up in our laboratory. Due to the retrospective nature of this study, no specific collection protocol was followed regarding time of specimen collection and dietary status. All patients (or their guardians) approved the possible use of their remaining samples for method validation purposes, in agreement with institutional and national legislation. IEM diagnosis was confirmed by enzymatic and genetic testing, when appropriate, according to available guidelines or expert opinion of the laboratory specialist or attending clinician. Control samples were obtained from remaining material from the general clinical chemistry diagnostic laboratory, again with patient approval. All samples were stored in a digital-alarm-controlled freezer at −20 °C before analysis for a period ranging from 3 years to 2 months. See Supplemental Table 1 for complete IEM diagnostic panel used for targeted evaluation of NGMS data.

**Table 1:**
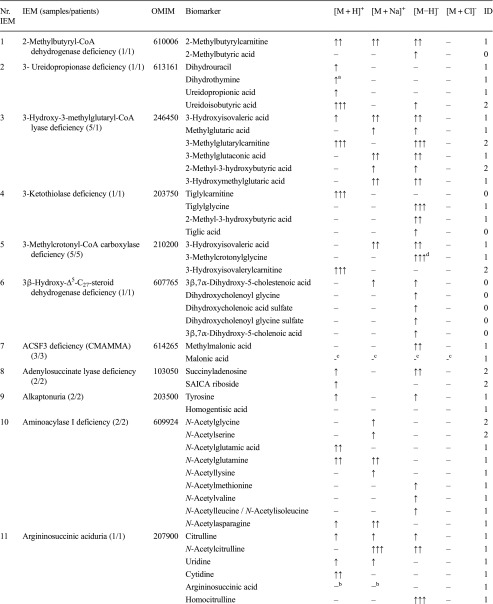

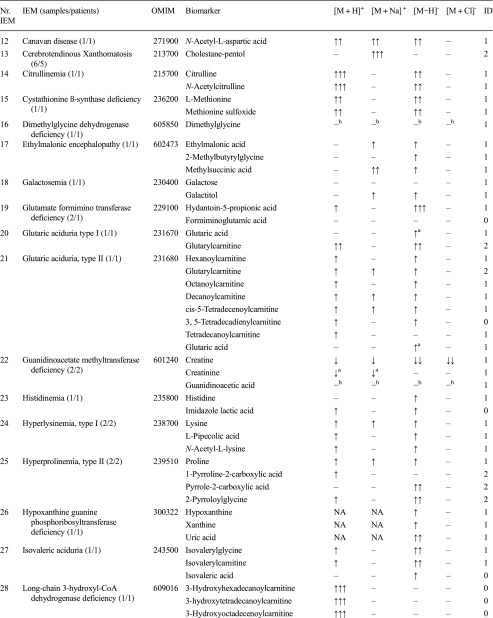

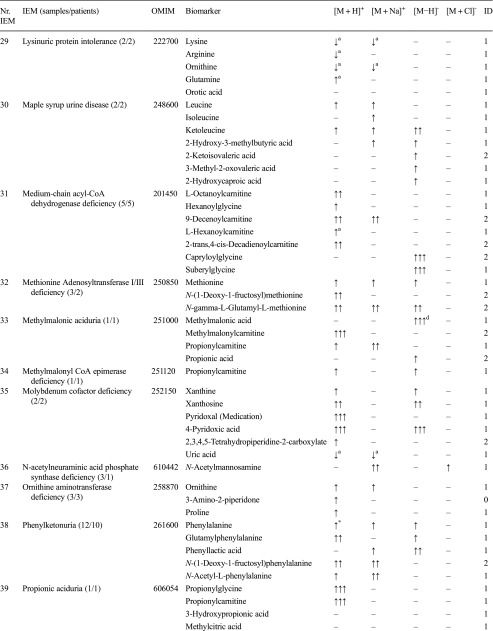

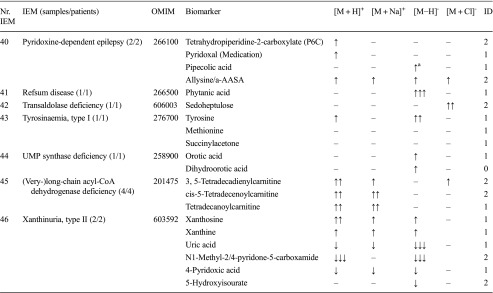
Overview of next-generation metabolic screening (NGMS) results for 46 distinct inborn errors of metabolism (IEMs). For each IEM, indicative metabolite alterations are shown. Please refer to Supplemental Table 1 for the complete IEM diagnostic panel used for targeted evaluation of NGMS data

Metabolite-related features were found in positive ([M + H]^+^ and [M + Na]^+^ adducts) and negative ([M − H]^+^ and [M + Cl]^-^ adducts) ionization modes

Arrows indicate increased (↑) or decreased (↓) intensity of the indicated feature in the patient sample compared with controls, one arrow: fold change 1–10, two arrows: fold change 10–100, three arrows: fold change >100. The column labeled ID indicates whether identity of a specific metabolite was confirmed by a model compound/reference standard (1), measurement of corresponding m/z in ≥2 diagnostic samples (2), or measurement of a corresponding m/z in a single diagnostic sample (0), NA indicates that the specific data was not available

^a^ Not selected by standard statistical testing, corrected *P* value >0.05

^b^Observed and abnormal in raw data but not observed in aligned data

^c^Not observed in raw data

^d^Identification based on ^13^C–isotope of feature due to saturation of ^12^C–isotope peak

### Sample preparation

Frozen human heparin-anticoagulated plasma was thawed at 4 °C and mixed by vortexing. An aliquot of 100 μl of plasma was transferred into a 1.5-ml polypropylene microcentrifuge tube. Then, 400 μl ice-cold methanol/ethanol (50:50 vol/vol) containing five internal standards (IS) [caffeine-d3 0.88 μmol/L, hippuric-d5 acid 0.22 μmol/L, nicotinic-d4 acid 0.88 μmol/L, octanoyl-L-carnitine-d3 0.22 μmol/L, L-phenyl-d5-alanine 0.44 μmol/L (all from C/D/N Isotopes, Pointe-Claire, Canada)] was added to each plasma aliquot. Samples were thoroughly mixed on a vortex mixer for 30 s, incubated at 4 °C for 20 min, and centrifuged at 18,600 g for 15 min at 4 °C. An aliquot of 350 μl of the supernatant was transferred into a 1.5-ml polypropylene microcentrifuge tube. Samples were dried in a centrifugal vacuum evaporator (Eppendorf, Hamburg, Germany) at room temperature, reconstituted in 100 μl of water containing 0.1% (vol/vol) formic acid, vortexed for 15 s, and centrifuged at 18,600 g for 15 min at room temperature. An aliquot of 90 μl was transferred into 250 -μl polypropylene autosampler vials. These samples were either placed in an autosampler at 4 °C for direct analysis or stored at −80 °C. Stored samples were thawed at room temperature and centrifuged at 18,600 g for 15 min at room temperature before analysis.

### UHPLC-QTOF-MS analysis

An Agilent (Santa Clara, CA, USA) 1290 ultra-high-performance (UHP) LC system coupled to an Agilent 6540 or 6545 QTOF mass spectrometer equipped with a dual electrospray ionization (ESI) source was used for untargeted analysis of plasma samples. Each sample was run in duplicate in both positive and negative ionization modes. A 2.0 -μl aliquot of extracted plasma sample was injected onto an Acquity HSS T3 (C18, 2.1 × 100 mm, 1.8 μm) column (Waters, Milford, MA, USA) operating at 40 °C. Chromatographic separations were performed by applying a binary mobile phase system. For analyses carried out in the positive ESI mode, mobile phase A consisted of water containing 0.1% (vol/vol) formic acid and mobile phase B was water/methanol (1:99 vol/vol) containing 0.1% (vol/vol) formic acid. For analyses performed in the negative ESI mode, mobile phase A consisted of water containing 10 mmol/L acetic acid and mobile phase B was water/methanol (1:99 vol/vol) containing 10 mmol/L acetic acid. The flow rate was 0.4 ml/min; for gradient elution, an isocratic period of 1 min at 1% B followed by a linear gradient from 1% B to 100% B over 15 min was applied. The final composition of 100% B was held constant for 4 min followed, by a return to 1% B in 1 min and an equilibration at 1% B for 4 min.

QTOF mass spectrometer was operated in positive ion mode with a capillary voltage of 2000 V, a nebulizer gas pressure of 60 psi, a drying gas temperature of 275 °C, and a drying gas flow rate of 11 L/min. For the negative mode, these characteristics were 4000 V, 35 psi, 250 °C, and 12 L/min. The mass spectrometer was operated in the extended dynamic range mode. Mass spectral data were acquired in the profile mode using a scan range of 60–1000 m/z with a scan time of 0.33 s.

Each analytical batch was composed of control plasma samples, IEM patient plasma samples, quality control (QC) plasma pool samples, a performance-check solution (PC), and a solution of IS. An analytical run consisted of a maximum of 150 samples. The set of control plasma samples was optimized for each run to mimic age- and gender variation in patient samples analyzed in that run. Minimum amount of control plasma samples in a single run was 15. To correct for possible run-order influence on signal intensities, duplicates of patient samples were analyzed in antiparallel run order, meaning that duplicates of the first patient sample were analyzed on first and last position in the analytical run, while duplicates of the last patient sample were analyzed in the two middle positions of the run. Also, in some runs, solutions containing a combination of reference standard compounds were subjected to NGMS analysis to establish retention time for identification purposes (see Supplemental Table 1 for specification of which reference standards were analyzed). These standards were acquired through several suppliers (Sigma, Brunschwig, Merck, J.T. Baker Chemical, Fluka Chemika, Cambridge Isotope Laboratories Inc., BDH, and Aldrich). The QC plasma pool consisted of a mixture of 800 plasma samples collected from leftover material from the clinical chemistry laboratory. Samples were selected from 50% male and 50% female patients varying in age between 1 month and 90 years; 50 μl of each individual sample was used to prepare the pool. The QC plasma pool was thoroughly mixed, and aliquots stored at −80 °C. The PC solution for the positive ESI mode was adenosine, caffeine, creatine, dimetridazole, epitestosterone, 2-methylbutyrylcarnitine, nicotinic acid, propionylcarnitine, stearoylcarnitine, L-tryptophan, and L-tyrosine in mobile phase A. Concentration of each compound was 0.1 μg/ml. The PC solution for the negative ESI mode was 2-amino − 3-methylbenzoic acid, trans-cinnamic acid, hippuric acid, 4-hydroxybenzoic acid, 12-hydroxyoctadecanoic acid, nicotinic acid, sebacic acid, L-tryptophan, L-tyrosine, and xanthine in mobile phase A. The concentration of each compound was 0.5 μg/ml.

Control and patient plasma samples were analyzed in duplicate. Eight random control plasma samples were injected at the start of each analytical batch to condition the analytical platform. The PC solution and the QC plasma pool were injected following 15–20 control and/or patient plasma samples. Data analysis of the PC solution and the QC plasma pool were used to monitor the performance of the analytical platform by calculating the technical precision within each analytical batch (within-run precision) for retention time (RT) and MS intensity. Precision data was based on data of all standards present in the PC solution, while for the QC plasma pool, a representative selection of endogenous metabolites was assessed: for the positive mode, piperidone, pyroglutamic acid, creatine, hypoxanthine, carnitine, uric acid, arginine, hippuric acid, homocitrulline, caffeine, propionyl-carnitine, sulfamethoxazol, hexanoyl-carnitine, octanoyl-carnitine, guanosine, and C18:1- and C18:2-lysophosphatidylcholine were evaluated, while in the negative mode, selection of metabolites included 2- and 3-hydroxy-butanoic acid, oxovaleric acid, 3-hydroxy-isovaleric acid, 3- and 4-methyl-2-oxovaleric acid, N-acetylalanine, xanthine, phenylalanine, phenyllactic acid, uric acid, hippuric acid, pyridoxic acid, hydroxyl-hippuric acid, sebacic acid, tryptophan, N-phenylacetylglutamine, inosine, testosterone sulphate, taurocholinic acid, and C16:0-, C18:0-, C18:2-, and C20:4-lysophosphatidylcholine.

### Data processing and statistics

The output files of the QTOF runs were aligned using the open-access software package various forms of chromatography mass spectrometry (XCMS) in single job modus. Following alignment and feature extraction, features were annotated against the Human Metabolome Database [HMDB] (Wishart et al. 2007) for putative metabolite identification. For all features, two-sided* t *tests were performed to identify significantly altered features between an individual patient and controls. Because of the large number of features identified in an individual patient (~10,000), the Bonferroni procedure (Dunn 1961) was used to correct for multiple testing to prevent false-positive selections, i.e., features incorrectly marked as significantly different in a patient. Two types of* t* tests were applied to compare the intensity of each feature present in an individual patient sample to the intensities observed in control samples. In the first test, replicate measurements of each observation (patient or control) were first averaged. Subsequently, for each feature present in an individual patient sample, the average intensity of its replicate measurements was compared with the mean intensity of that feature in all control samples. In the second test, the single-patient measurement of a specific feature that was most similar to controls (i.e., one of two replicates) was used for comparison with means of control plasma samples. The Bonferroni procedure was applied separately to the output of the two types of* t* tests separately. Only peaks marked as significantly different by both approaches after Bonferroni correction (*P* value <0.05) were retained for further analysis. This combination of tests, rather than using a single* t* test, was employed to control for false positives in case of technical variability of the UHPLC-QTOF setup.

### Data interpretation

To extract relevant diagnostic information from the untargeted metabolomics data, we developed an in-house IEM panel that consisted of 340 IEM-related metabolites, which was used to filter the total list of identified features. Based on the exact mass of a metabolite, hypothetical masses of proton, sodium, and chlorine adducts were calculated and included in this IEM panel, as well as hypothetical masses for deprotonated ions. Additionally, hypothetical masses of C isotopes (Tebani et al. 2017) were calculated and incorporated. Information on established retention times of reference standards, which were available for 222 of the 340 panel metabolites (65%), was included in the IEM panel to allow for high confidence identification according to guidelines of the Metabolomics Standards Initiative (Sumner et al. 2007). For metabolites unavailable as reference standard, evidence for identification was gathered from biological reference samples (patients with established IEM diagnosis, for 36/340 panel metabolites, 11%), isotope ratios, specific in source fragmentation patterns, HMDB classification (endogenous versus drug, food, or microbial origin of metabolite), or Kyoto Encyclopedia of Genes and Genomes (KEGG) (Kanehisa et al. 2017) information on common metabolic pathways. Significantly different features were extracted based on the* t* test procedure described above, a ppm deviation of <5 for mass accuracy, and—when a reference standard was available—a relative retention time difference between this reference standard and the metabolite in the biological sample of <10%. Features that withstood this filtering procedure were then prioritized based on their intensity and on the fold difference in intensity between mean intensity of the patient feature and controls (fold change). Intensities of a specific feature, comparing an individual patient to all controls analyzed within one analytical run, were visualized in barplots using Unscrambler software (Camo Software, Oslo, Norway).

## Results

### Analytical performance

Samples were measured over 60 analytical series in which both positive and negative MS ionization modes were applied. The series were performed from November 2013 to May 2017. In July 2016, an upgrade of the QTOF system was implemented, which involved a switch from the Agilent 6540 to the Agilent 6545; the same UHPLC system was used with both. A selection of IEM patient samples, based on availability, was rerun on the upgraded setup as part of its validation procedure. In total, 20 of the 60 analytical runs described here were performed on the Agilent 6545 setup. There was no significant difference in the identification of clinically relevant metabolites between systems, confirming the reliability and robustness of the methodology.

In the validation, the Agilent 6545 showed increased sensitivity with a factor up to ten for different metabolites, as was expected from the manufacturer’s specifications. For other performance characteristics, the Agilent 6545 was either comparable with or superior to the Agilent 6540; therefore, only specifications of the Agilent 6545 analytical performance are further detailed here. Stability of the analytical platform was evaluated based on within-run precision data of the PC samples. For standards with retention times >2 min, a coefficient of variation (CV) of ≤0.5% for retention time was accepted, while for retention times <2 min, this limit was set to ≤1%. Results of within-run precision of retention times always fulfilled these criteria and in practice rarely exceeded 0.5%, as for retention times <2 min. For MS signal intensities detected for the standards, a maximum within-run CV of 15% was allowed, adhering to recommendations from the literature for performance (Dunn et al. 2011). The actual analytical within-run CV for intensity was lower than the target limit—mostly <10%. For mass accuracy, a maximal error of 5 ppm was accepted, but in practice, this rarely exceeded 3 ppm. Regarding between-run precision, a maximum CV of 1% was found for retention times >2 min and 2% for retention times <2 min. For MS signal intensities, the median of between-run CVs was 23% in the negative mode and 30% in the positive mode.

A plasma pool QC sample was included several times in each run to gain insight into within-run precision of retention time and intensity for a selection of endogenous metabolites in a clinically relevant matrix. For both positive and negative ionization modes, a specific combination of metabolites was selected for evaluation (see “Methods” section). Within-run precision performance criteria were equal to those for the PC sample, with the exception of the CV accepted for the MS signal intensity, which was 20%. Regarding between-run precision for the plasma pool QC sample, comparable performance with the PC sample was reached. CVs for retention time were <2%, while median CV for intensity was 16% for the negative and 31% for the positive mode.

To evaluate the correlation of semiquantitative NGMS results to the exact molar concentrations obtained through conventional techniques, we compared them for a selection of metabolites: phenylalanine, leucine, free carnitine, octanoylcarnitine, decenoylcarnitine, and 3-hydroxyisovalerylcarnitine. For most samples, conventional carnitine and amino acid analysis was performed, enabling this comparison. Linear regression analysis showed good correlation between intensities measured in NGMS and actual concentration, with, for example* R*^2^ values >0.95 for octanoyl-carnitine, 3-hydroxy-isovaleric carnitine, and phenylalanine. For free carnitine, correlation was least optimal, with an* R*^2^ of 0.5651 (Supplemental Fig. 1).

### NGMS-based screening of IEMs in individual patients

**Fig. 1:**
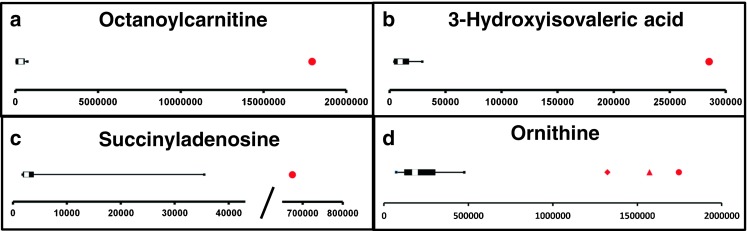
Feature intensity for a selection of inborn errors of metabolism (IEMs.) In all panels, boxplots show feature intensity distribution in control plasma samples, the* X-axis* represents feature peak area in arbitrary units. The* black box *represents the middle 50% of the distribution in control plasma samples;* white square *represents median of this distribution; left and right whiskers represent lowest and highest value measured in controls. Patient values are shown in* red*.** a** Medium-chain acyl-CoA dehydrogenase deficiency: data shown for octanoylcarnitine, m/z 288.2179 ([M + H] ^+^ adduct, retention time (RT) 9.60 min), which is significantly increased in the patient sample compared with 27 controls (fold change 52.8).** b** 3-Hydroxy-3-methylglutaryl CoA-lyase deficiency: data shown for 3-hydroxyisovaleric acid, m/z 117.0557 ([M − H] ^−^ adduct, RT 3.43 min), which is significantly increased in the patient sample compared with 28 controls (fold change 28.1).** c** Adenylosuccinate lyase deficiency: data shown for succinyladenosine, m/z 384;1144 ([M + H]^+^ adduct, RT 4.71 min), which is significantly increased in the patient sample compared with 29 controls (fold change 264.6).** d** Ornithine amino transferase deficiency: data shown for ornithine, m/z 133.09714 ([M + H]^+^ adduct, RT 0.49 min), which is significantly increased in three patient samples compared with 27 controls (mean fold change 7.1)

We applied our NGMS technology to delineate plasma metabolomic profiles of 46 distinct IEMs (Table 1). All patients were on diagnosis-specific treatment following available guidelines or based on expert opinion of attending clinicians. Because of treatment, biochemical abnormalities were expected to be less pronounced compared with those detected in samples of untreated patients. In each individual plasma sample, >10,000 features could be detected. Due to the high level of complexity of data generated through this untargeted NGMS methodology, we searched for an application feasible for future implementation in routine patient diagnostics. In analogy to routine diagnostic analysis of big data generated from next-generation (exome) sequencing strategies through disease-specific filters, we developed an IEM metabolite panel containing 340 clinically relevant metabolites known to be either diagnostic or associated with common therapeutic interventions for IEMs (Supplemental Table 1) as a first filtering step to reduce data complexity and extract relevant diagnostic information. Through a semi-automated pipeline developed in-house, all features detected in a plasma sample were compared between an individual patient and controls. Bonferroni-corrected *P* values were calculated for statistical significance of each feature. Open-access metabolite information from the HMDB was integrated for metabolite annotation (see “Methods” section). Only significantly different features between a patient and controls were evaluated and reported. Based on chemical characteristics of the metabolite, different adducts were preferentially formed (see Table 1). Following this approach, we identified key diagnostic metabolites in 42 of 46 (91%) IEMs tested (see Fig. 1) to visualize results in barplots of feature intensity for a selection of IEMs. Among those IEMs were several organic acidurias, for which the relevant urinary biomarkers could readily be detected in plasma using the NGMS approach. In our set of 46 IEMs tested, four diagnoses remained undetected by the standard NGMS workflow: guanidinoacetate methyltransferase (GAMT) deficiency, argininosuccinic aciduria, dimethylglycine dehydrogenase deficiency, and lysinuric protein intolerance. In the first three of these diseases, peaks could be detected in raw data files at the expected m/z and retention time for the relevant metabolites guanidinoacetate, argininosuccinic acid, and dimethylglycine (Supplemental Fig. 2). However, these features were not present in the XCMS output file, indicating that they were lost in the alignment procedure. For lysinuric protein intolerance, a negative fold change (−3.2×) for lysine was observed in raw data but was rejected in our statistical selection (corrected *P* value >0.05).

For saturated peaks, feature annotation can fail if the required level of mass accuracy cannot be reached. Saturation was found for phenylalanine in phenylketonuria patients and for 3-methyl-crotonylglycine in 3-methyl-crotonyl-CoA carboxylase (3-MCC) deficiency. To overcome this issue, we used the unsaturated peak of the naturally occurring ^13^C isotope for identification. This approach led to successful identification of phenylalanine and 3-methyl-crotonylglycine as being significantly increased in relevant patient samples, despite saturation of the ^12^C signal. To prevent any possible false negatives due to peak saturation of the respective biomarker, we included the calculated hypothetical masses of the ^13^C isotopes of all metabolites in our IEM reference panel.

To test the robustness of our NGMS approach, we applied this method to a batch of ten samples (hyperprolinemia type II, hyperlysinemia type I, maple syrup urine disease (MSUD), aminoacylase I deficiency, and 3-hydroxy−3-methylglutaryl-CoA lyase deficiency; two samples for each disease) in a blinded fashion, meaning that both the technician and the laboratory specialist interpreting the metabolomic profile had no prior knowledge of the diseases selected. Through our NGMS pipeline, all five disorders could be unequivocally diagnosed through the expected metabolic perturbations, as stated in Table 1 (data for blinded study are not shown separately).

### Added clinical value of NGMS-based diagnostics in IEMs

**Fig. 2:**
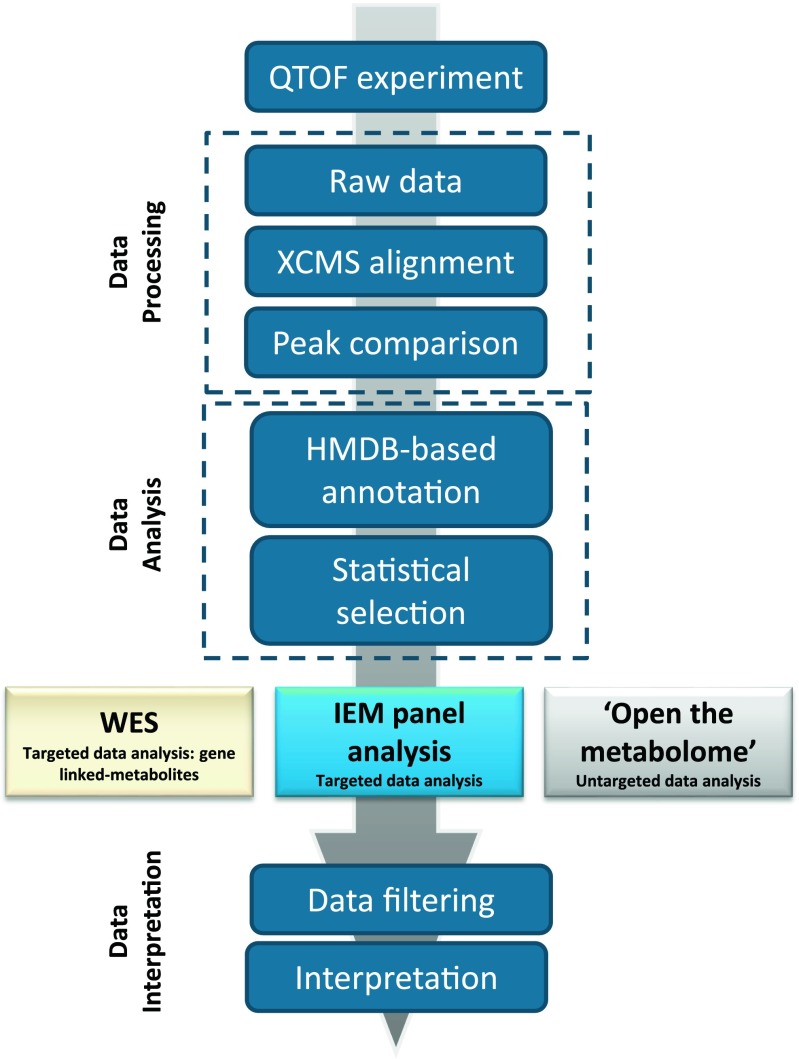
Next-generation metabolic screening (NGMS) multistep workflow

Having shown that NGMS is capable of diagnosing a broad spectrum of known IEMs, we now highlight novel applications of this methodology that provide additional clinical value compared with conventional biochemical IEM screening. We present a multistep NGMS workflow in which three main pillars for data analysis can be defined (Fig. 2): besides targeted IEM panel analysis, whole-exome sequencing-directed analysis and untargeted analysis (open the metabolome) can be performed. We illustrate these pillars through a selection of cases.

#### Targeted IEM panel analysis: increased specificity for diagnosis of xanthinuria types I and II through plasma NGMS analysis

**Fig. 3:**
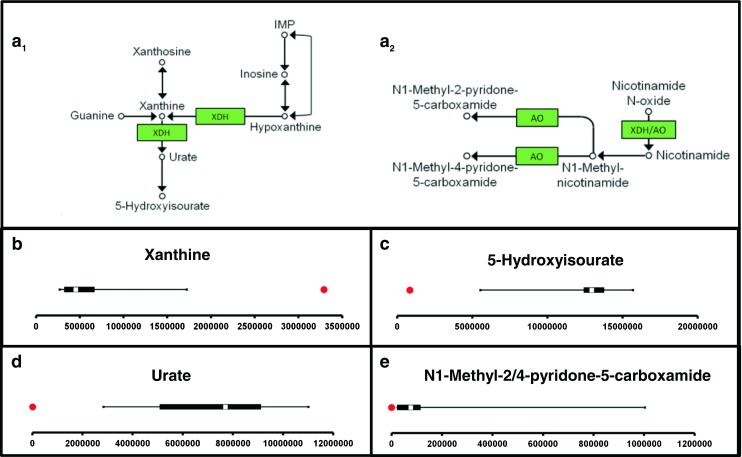
Next-generation metabolic screening (NGMS) results in xanthinuria type II. Panels b–e, show the feature intensity distribution in 26 control plasma samples (*X-axis* represents feature peak area in arbitrary units), and should be interpreted as described in Fig. 1.* Red* patient values.** a** xanthine dehydrogenase (XDH) (**a1**) and aldehyde oxidase (AO) function (**a2**).** b** Xanthine, m/z 151.02624 ([M − H] ^−^ adduct), retention time (RT) 2.02, is significantly increased in the patient sample (fold change 7.2).** c** 5-Hydroxyisourate, m/z 183.01634 ([M − H]^−^ adduct), RT 1.47, is significantly decreased in the patient sample (fold change −15.4).** d** Urate, m/z 167.02137 ([M − H]^−^ adduct), RT 1.49, is virtually absent in the patient sample (fold change −810.2).** b–d** represent perturbations resulting from defective xanthine dehydrogenase (XDH) function (see pathway in** a1**).** e** N-1-methyl-4/2-pyridone-5-carboxamide, m/z 153.0659 ([M + H] ^+^ adduct), RT 2.95, is virtually absent in the patient sample (fold change −232.4) due to defective AO function (see pathway in** a2**), and results are therefore indicative of type II

Identifying and validating diagnostic biomarkers are key objectives to support IEM screening diagnostics. In 2012, Peretz et al. reported novel urinary biomarkers that can distinguish between xanthinuria type I [isolated xanthine dehydrogenase (XDH) deficiency] and type II [combined deficiency of XDH and aldehyde oxidase (AO)] (Peretz et al. 2012). These specific markers for type II are a result of AO deficiency in several metabolic pathways: increased N-methylnicotinamide and decreased N-methylpyridone carboxamides from the nicotinamide degradation pathway; decreased hydantoin propionic acid from histidine metabolism; decreased N1-methyl-8-oxoguanine from nucleic acid metabolism; and decreased pyrrolidine-2-one from spermidine degradation. Using our NGMS strategy, we evaluated these AO-specific urinary markers in plasma of type II patients and replicated urinary findings in plasma, in which we found even more pronounced fold changes between patient and controls than described by Peretz et al. (Fig. 3). These data show that our NGMS methodology is able to specifically diagnose xanthinuria type II in plasma. Remarkably, in contrast to the results of Peretz et al., we confirmed the involvement of AO in vitamin B6 metabolism, as we found a significantly decreased concentration of pyridoxate in patients compared with controls.

#### Whole-exome sequencing (WES)-directed evaluation of NGMS data

**Fig. 4:**
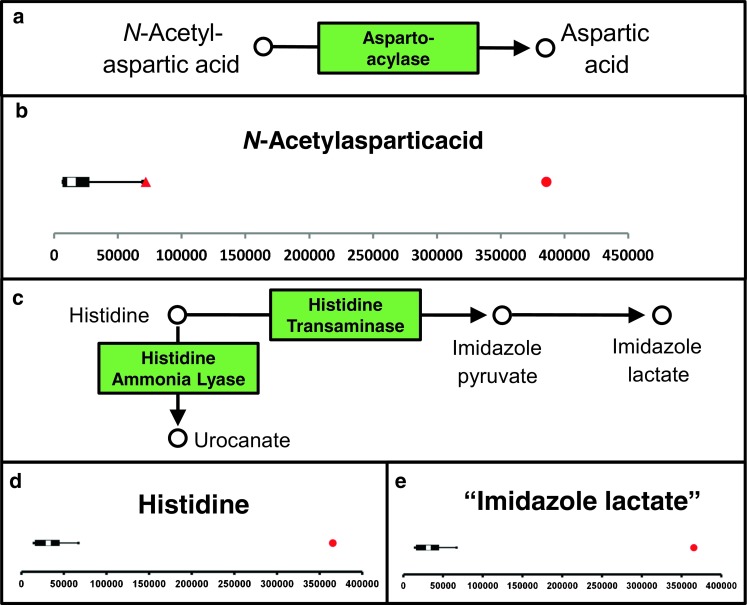
Next-generation metabolic screening (NGMS) for Canavan disease and histidinemia. In panels b, d, and e, boxplots show the feature-intensity distribution in control plasma samples (*N* = 27, *N* = 29, and* N* = 29, respectively; the X-axis represents feature peak area in arbitrary units) and should be interpreted as described in Fig. 1.* Red* patient values.** a** N-acetylaspartic acid metabolism: in Canavan disease, the function of aspartoacylase is deficient.** b** N-acetylaspartic acid [M + Na] ^+^ feature (m/z 198.03723, retention time (RT) 1.15): significantly increased in plasma (*red circle*, fold change 36.5). In a patient with a variant of uncertain significance (VUS) in the Canavan-associated* ASPA* gene (*red triangle*), this feature was not significantly altered (fold change 4.6).** c** Histidine metabolism: in histidinemia, the function of histidine ammonia lyase is deficient.** d** Histidine, m/z 154.06247 ([M − H] ^−^ adduct), RT 0.60, is significantly increased in the patient sample (fold change 6.9).** e** A feature with m/z 157.06074 and RT 0.71: significantly increased in the patient sample (fold change 13.6); this feature putatively represents the [M + H] ^+^ adduct of imidazole lactic acid, which is derived from metabolism of accumulating histidine, as depicted in** c**

For variants of uncertain significance (VUS) identified in metabolism-related genes, NGMS can provide a functional counterpart to further delineate the possible pathogenic nature of the genetic alteration. As an example, in a 1-year-old patient with developmental delay, epilepsy, and hearing loss, a homozygous missense variant of uncertain significance [c.509 T > C; p.(Ile170Thr)] was identified in *ASPA* (NM_001128085.1) through whole-exome sequencing. At the time of ASPA Ile170Thr variant identification in our patient, this variant had not been described before, and its pathogenicity was considered to be unclear based on modest conservation of the mutated amino acid and presence of the variant in the heterozygous state in control cohorts (0.10% in GoNL and 0.031% in The Exome Aggregation Consortium). Mutations in *ASPA* are associated with Canavan disease (OMIM 271900), a leukodystrophy that presents in the first year of life with delayed psychomotor development, hypotonia, macrocephaly, and epilepsy after an initially normal development. In the analysis of organic acids in urine of this patient, a mildly increased excretion of the Canavan-linked metabolite N-acetylaspartate was found (~200 μmol/mmol creatinine); however, in other known Canavan patients, a more pronounced excretion of N-acetyl aspartate (>1000 μmol/mmol creatinine) is usually detected. Therefore, organic acid analysis in urine did not confirm nor exclude the diagnosis. In the Canavan patient included in our NGMS clinical validation, a significantly increased concentration of N-acetylaspartate could readily be detected in plasma (Table 1, IEM 12). Following NGMS analysis, for the patient with the ASPA Ile170Thr VUS, we found N-acetylaspartate in plasma was in the high–normal range but not significantly altered compared with controls (Fig. 4a, b). Therefore, we propose that the ASPA Ile170Thr VUS likely retains residual enzymatic activity, making it nonpathogenic in homozygous form.

#### Untargeted open the metabolome analysis

In the pillar of untargeted NGMS analysis, two modalities can be defined: identification of novel biomarkers in known IEMs, and identification of as yet unknown IEMs. As an example of the first modality, untargeted analysis of the known IEM histidinemia (OMIM# 235800) showed a significantly increased concentration of a feature with an m/z of 157.0768 and RT of 0.71 min in positive ionization mode (Fig. 4e). According to HMDB annotation, this mass could correspond to the protonated adduct of imidazole lactic acid (HMDB 02320). According to information in KEGG, imidazole lactic acid can be formed nonenzymatically from imidazole pyruvate, which is in its turn formed from histidine by histidine transaminase. Accumulation of histidine is therefore likely to lead to an increased concentration of imidazole lactic acid. This metabolic perturbation has not been reported previously in histidinemia but is now putatively detected by our NGMS approach, even though definite identification will require further study of a model compound or orthogonal methods, such as NMR, MS/MS, or infrared spectroscopy. Also, we performed open the metabolome analysis in seven PKU patients under treatment. It was apparent that, besides previously described PKU biomarkers, two unknown features with m/z 328.1391 (RT 4.01 min) and 424.1716 (RT 6.38 min) were identified as significantly increased in most patients. Very recently, these features were identified using multistage fragmentation MS as a phenylalanine–hexose conjugate and a glutamine-glutamine-phenylalanine tripeptide, respectively (Vaclavik et al. 2017). These novel markers showed higher interpatient variation compared with the regular marker, phenylalanine, making them interesting candidates to further explore predictive value for patient clinical status—for example, for the effectiveness of dietary intervention. The second open the metabolome modality encompasses identifying as-yet unknown IEMs through novel biomarkers. Untargeted data analysis can identify novel markers that, in combination with information from KEGG and/or WES analysis, can be linked to disease. An example of this was the identification of a significantly increased feature in body fluids of patients with severe developmental delay and dysmorphism, which could be annotated as N-acetylmannosamine. This finding led to the novel diagnosis of N-acetylneuraminic acid phosphate synthase (NANS) deficiency, as was previously reported by us (van Karnebeek et al. 2016).

## Discussion

We here present NGMS as a single-platform, untargeted, high-resolution LC-QTOF, metabolomic profiling method that can be applied in the diagnostic screening for IEMs in individual patients. We were able to show the capability of the NGMS setup for the diagnosis of 46 individual IEMs through relevant biomarkers. The strength of our NGMS workflow is that, even though targeted screening for known IEM-associated metabolites is performed as a first step, untargeted metabolomics data is available to undergo a subsequent round of untargeted data analysis, which we term “open the metabolome”. We foresee a workflow in which unclassifiable perturbations in IEM panel analysis or negative results for highly suspect patients will be followed up by untargeted data analysis. Also, patients already diagnosed with an IEM but with an atypical response to treatment or disease course are relevant candidates for untargeted NGMS analysis. This stepwise strategy will allow for the identification of novel biomarkers and diseases while containing manageability of NGMS data for routine IEM diagnostics. The stepwise NGMS strategy we present here is comparable with the approach taken in the genomics field to whole-exome sequencing analysis. In the current-day situation, whole-exome analysis is mostly initiated with targeted evaluation of a disease-related selection of genes, which can subsequently be expanded to opening all exome data. This strategy has proven its effectiveness in clinical diagnostics and in the identification of novel disease-causing genes (Mendes et al. 2017; Miller et al. 2015). An ideal workflow would be to perform genomics and metabolomics analysis concurrently, with the goal of providing a functional context to interpret genetic variants of uncertain significance. Previous studies have been performed showing the complementary nature of genomic and metabolomic analyses for interpreting genetic variants (Rhee et al. 2016; Guo et al. 2015; Long et al. 2017; Pappan et al. 2017). Combining genomics and metabolomics data has also led to the discovery of diagnostic biomarkers or diagnostic-biomarker fingerprints for genetic diseases (Dunn 1961; Sumner et al. 2007; Wishart et al. 2007; Dunn et al 2011; de Ligt et al. 2012; Gilissen et al. 2012; Guo et al. 2015; Miller et al 2015; Abela et al. 2016; Rhee et al. 2016; van Karnebeek et al. 2016; Abela et al. 2017; Kanehisa et al. 2017; Long et al. 2017; Pappan et al. 2017; Vaclavik et al. 2017). Additionally, further integration of metabolomics and genomics with phenomics data, making use of the specialized expertise of clinicians and laboratory specialists, has shown great potential, as described by Tarailo-Graovac et al. (2016).

A first proof of the diagnostic power of our NGMS setup in combination with genomics data was obtained through the discovery of a novel IEM: NANS deficiency (van Karnebeek et al. 2016). As a second example, in this article, we present a variant of uncertain significance in the gene associated with Canavan disease (*ASPA*), which according to our NGMS data suggest not to be disease-causing in the homozygous form. During preparation of this manuscript, the ASPA Ile170Thr variant was reported in a study that correlated residual aspartoacylase enzyme activity to patient geno- and phenotype (Mendes et al. 2017). In their study, the Ile170Thr variant was reported to be homozygous in another patient with a mild phenotype not typical for Canavan disease. In an in vitro enzyme-activity assay in transfected HEK239 cells, a relatively high residual activity was found for the Ile170Thr variant. The conclusion of the authors was therefore that Ile170Thr is a rare variant of uncertain clinical significance. Our NGMS results in plasma now further support these findings. In all likelihood, the signs and symptoms of our patient must have another underlying cause. We cannot exclude, however, that the ASPA Ile170Thr variant in combination with a nonsense variant would cause a classical Canavan phenotype.

Even though the preceding data perfectly illustrate the promises that NGMS holds for the field of IEM diagnostics, some challenges encountered during the validation of our NGMS method need to be addressed. As was described in the “Results” section, for four of 46 IEMs tested, diagnosis could not be established through our standard NGMS workflow. Two main reasons for false-negative results could be defined. First, some metabolites were not recognized by the XCMS alignment algorithm, as they were not identified in the aligned data files, while inspection of the raw data did confirm perturbations in their levels compared with controls. These alignment issues arose for guanidinoacetate, argininosuccinic acid, and dimethylglycine, which all had a short retention time of ~0.6 min, which is still considered to be in the void volume of the UHPLC column. In general, polar metabolites, such as amino acids and sugars, exhibit only marginal retention on reverse-phase columns, as used in the NGMS setup described here. However, the relatively short retention time did not appear to be the major reason for alignment failure, as for several other metabolites that co-eluted ~0.6 min (e.g., citrulline, sedoheptulose, and methionine sulfoxide), the alignment procedure was correct. The fold changes of missing and correctly aligned metabolites with a retention time of ~0.6 min were of comparable range. The exact cause of the failure of the XCMS algorithm for alignment of guanidinoacetate, argininosuccinic acid, and dimethylglycine is therefore as yet unclear. In future development of our NGMS bioinformatic pipeline, we aim to develop an in-house feature-alignment algorithm that can be further tested and optimized to prevent alignment errors. Also, the application of a second column type, such as hydrophilic liquid interaction chromatography (HILIC) (Cuykx et al. 2017), will improve retention times for polar compounds and reduce co-elution of these metabolites, which will allow for optimal resolution of peaks and likely facilitate their correct alignment.

Another marker that was not detected by the alignment algorithm was alloisoleucine. In the MSUD patients tested in this study, no alloisoleucine was reported as significantly increased in the final NGMS results. Looking retrospectively at the raw UHPLC-QTOF-MS data, we did observe a dual peak at the position of isoleucine (Supplemental Fig. 3). Upon analysis of a mixture of the model compounds of alloisoleucine and isoleucine, a similar peak pattern was observed as for the MSUD patients, likely confirming the presence of alloisoleucine. However, it is clear that these stereoisomers cannot completely be separated on the UHPLC column, and in the alignment, these peaks are clustered, leading to a single annotation of isoleucine, which stands out as significantly increased compared with controls. As other specific MSUD biomarkers were identified as significantly disrupted in the NGMS analysis (such as 2-hydroxy−3-methylbutyric acid and 2-hydroxyisocaproic acid, see Table 1), the missing alloisoleucine identification did not cause a false-negative result for MSUD.

A second cause of unsuccessful metabolite identification can be sought in the very strict statistical selection procedure of significantly different features between an individual patient and controls. As >10,000 features are found in each sample, statistical comparison of features between samples should correct for false-positive identification due to multiple testing. To overcome this issue, we made use of the Bonferroni correction, which is the most stringent multiple testing correction. It divides the overall desired *P* value for significance by the amount of* t* tests performed, thereby controlling the probability that at least one* t* test will give a false-positive result. Because of this strict statistical selection, modest fold changes between patient and controls might be missed, or high variation in MS signal intensity between control samples could lead to incorrect statistical dismissal. We see an example of this for the patient with lysinuric protein intolerance (on benzoate treatment). No relevant metabolite disruptions (including decreased lysine or increased orotic acid concentrations) were detected in the NGMS analysis in plasma. One might speculate that in urine, significantly increased lysine, ornithine, and/or arginine may have been identified; however, we did not yet apply our NGMS approach to urine samples. Upon evaluation of the raw NGMS data for lysine, a negative fold change was observed. In the conventional amino acid analysis using ion-exchange chromatography, the lysine concentration was clearly decreased—37 µmol/L, with a lower reference limit of 81. However, in the NGMS data-processing pipeline, the lysine-associated feature was rejected in the statistical selection, as the corrected *P* value was >0.05. With the analysis of big data, such as our NGMS results, the challenge lies in finding the right balance between reducing false-positive identifications while preventing false negatives. In our strict correction procedure, false positives are nearly excluded, but there is a risk for false negatives for relatively mild perturbations and/or features that show a high variation in controls. In an update of our NGMS data-processing pipeline, we intend to evaluate the less strict Benjamini–Hochberg false discovery rate correction (Benjamini and Hochberg 1995). The main difference is that the Benjamini–Hochberg procedure is designed to control the expected proportion of false-positive discoveries in a final set of significantly disrupted features, so with a* P* value of <0.5, maximally 5% of identified features can be false positives. In our validation study, we mainly used samples of patients who were previously diagnosed and who already received appropriate treatment for their specific condition. This clinical management could alleviate the biochemical perturbations in these patients, and one could argue that in screening samples of yet undiagnosed patients, metabolite alterations will be more pronounced, and strict statistical selection would therefore be less of an issue. We will also evaluate multivariate approaches to identifying significant metabolite aberrations (Engel et al. 2014, 2017). Our goal in this would be to automatically map multiple metabolite perturbations on a single, most likely perturbed, metabolic pathway and to take information of shared pathways into account for determining statistical probability in identification. We are currently expanding our bioinformatics pipeline to include pathway information from KEGG (Kanehisa et al. 2017) and ReconMap 2 (Noronha et al. 2017; Thiele et al. 2013) to realize this next level in NGMS data processing.

We tested our NGMS methodology in a substantial set of 46 different IEMs. Due to the rare nature of this class of diseases, it is impossible to directly test diagnostic efficiency of NGMS in patient plasma for all known IEMs. However, based on a recently published IEM database containing clinical, biochemical, and phenotypic profiles of 530 known IEMs (IEMbase; Lee et al. 2017), we made an educated estimation of the proportion of IEMs that could hypothetically be detected by our approach. We cross-referenced the 340 metabolites of our IEM panel to IEMbase through their HMDB IDs and extracted the OMIM codes of their associated IEMs. Based on this comparison, we could extrapolate the diagnostic yield of our IEM panel metabolites and estimate that at least 205 individual IEMs can be diagnosed by our single-platform NGMS setup. IEMs that are not covered in the current situation include lysosomal storage disorders, congenital disorders of glycosylation, disorders of steroid metabolism, disorders of (apo)lipoprotein metabolism, porphyrias, and disorders of copper metabolism as main disease categories. When a patient’s phenotype is suggestive for one of these diseases, conventional targeted biochemical analysis should be performed in parallel to NGMS. Additionally, lipidomics (Griffiths et al. 2011) could be applied as a complementary holistic lipid-screening method alongside our NGMS methodology. Finally, after a diagnosis has been made, a dedicated targeted method may be applied to provide quantitative information on relevant disease biomarkers for patient follow-up.

As our IEM panel strategy relies on identifying metabolites through their unique HMDB identifier, known disease metabolites not yet available in HMDB might be missed. To prevent this issue, we set up a close collaboration with HMDB to ensure addition of missing endogenous metabolites to HMDB. For the subsequent step of untargeted open the metabolome analysis, apart from identification based on HMDB, we will evaluate unknown features when significantly altered in a patient through other databases, such as Metlin or KEGG. Significant features for which identification is not possible, which we term “features of uncertain significance” (FUS), will be stored in an in-house database coupled to anonymized patient phenotype and medication data for future reference. In every individual patient, many FUS are found upon untargeted analysis of significantly differing features. These FUS may be the result of modifications of metabolites in human endogenous metabolism; however, they may also derive from food, medication, or the intestinal microbiome, adding an extra layer of complexity to the metabolomic profile. The number of FUS in every plasma sample is considerable and a major hurdle that restricts the current clinical applicability of the open the metabolome strategy. This issue asks for concerted action of the IEM field to shed further light on this. When a FUS persists in multiple samples of an individual patient, is present in low frequency in the FUS database, and medication can be excluded as a source, it may be a relevant novel biomarker for a patient’s disease. Such apparent biomarkers can be selected for further investigation to establish identification, for example, through multistep fragmentation MS, by infrared spectroscopy (Martens et al. 2017), or via orthogonal approaches such as as NMR spectroscopy. Sharing FUS experience between centers will certainly advance the field further and guide the interpretation of untargeted metabolomics data.

In conclusion, we present a single-platform, untargeted, high-resolution, LC-QTOF, metabolomic profiling method—NGMS—which can be successfully applied for diagnosing a substantial spectrum of IEMs in individual patients. Through a dual targeted/untargeted data analysis strategy, we can achieve swift diagnosis of known IEMs while allowing for identification of novel biomarkers and diseases. As a third option, we can integrate genomics and metabolomics data to facilitate interpretation of genetic variants of uncertain significance. Even though challenges lie ahead in optimizing our methodology, we are convinced that metabolomics is the way forward for biochemical diagnostics in the field of IEMs.

## Electronic supplementary material

Supplemental Figure 1 (PPTX 642 kb)

Supplemental Figure 2 (PPTX 176 kb)

Supplemental Figure 3 (PPTX 131 kb)

Supplemental Table 1 (XLSX 32.9 kb)
