# Supplementary material for: Next-generation metabolic screening: targeted and untargeted metabolomics for the diagnosis of inborn errors of metabolism in individual patients
Source: J Inherit Metab Dis. 2018 Feb 16;41(3):337–53. doi: 10.1007/s10545-017-0131-6 (PMC5959972; doi:10.1007/s10545-017-0131-6)
Supplement: Supplementary file 1 — (PPTX 642 kb) [file 10545_2017_131_MOESM1_ESM.pptx]

## Slide 1
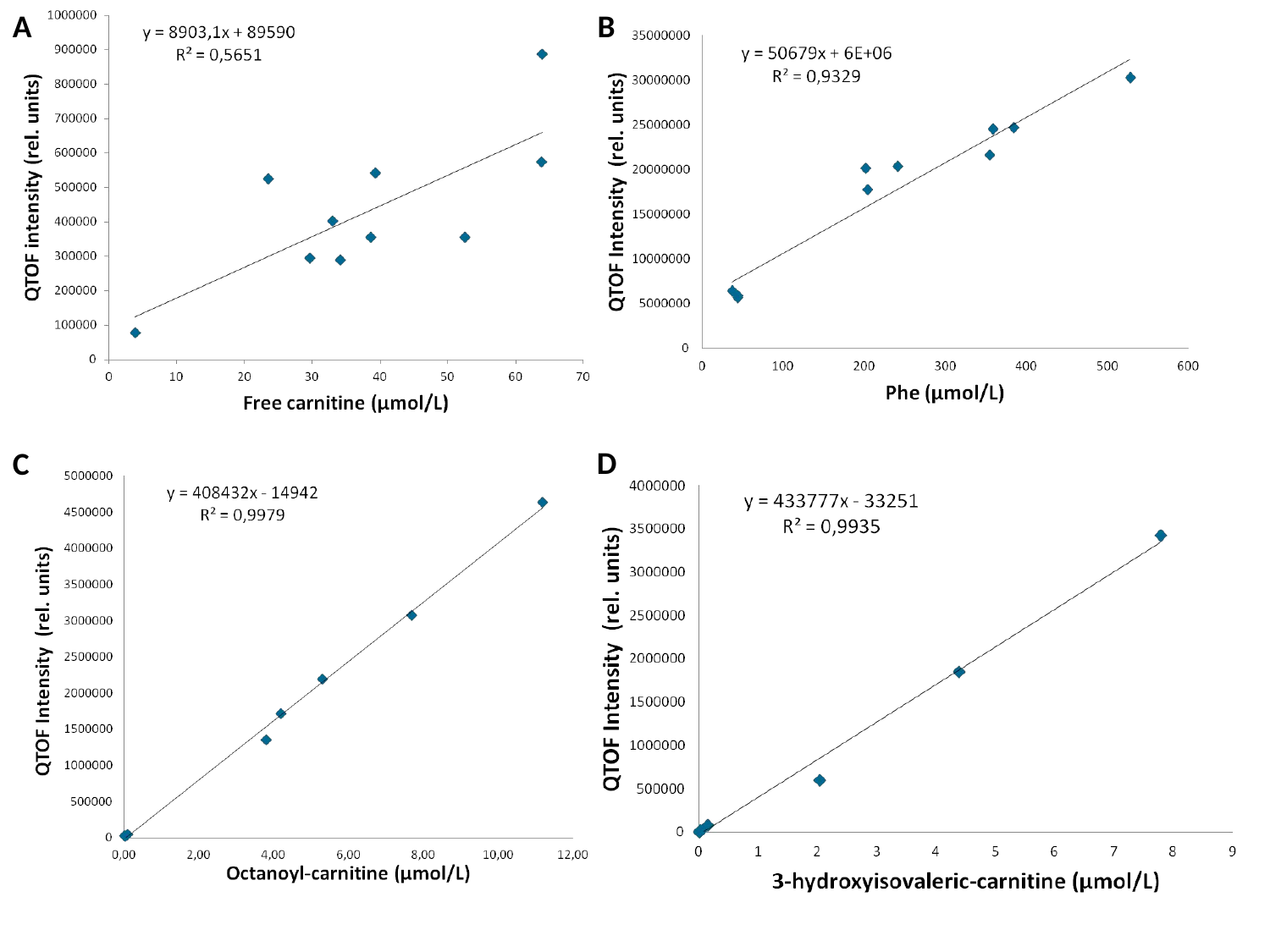

A
B
D
C

## Slide 2
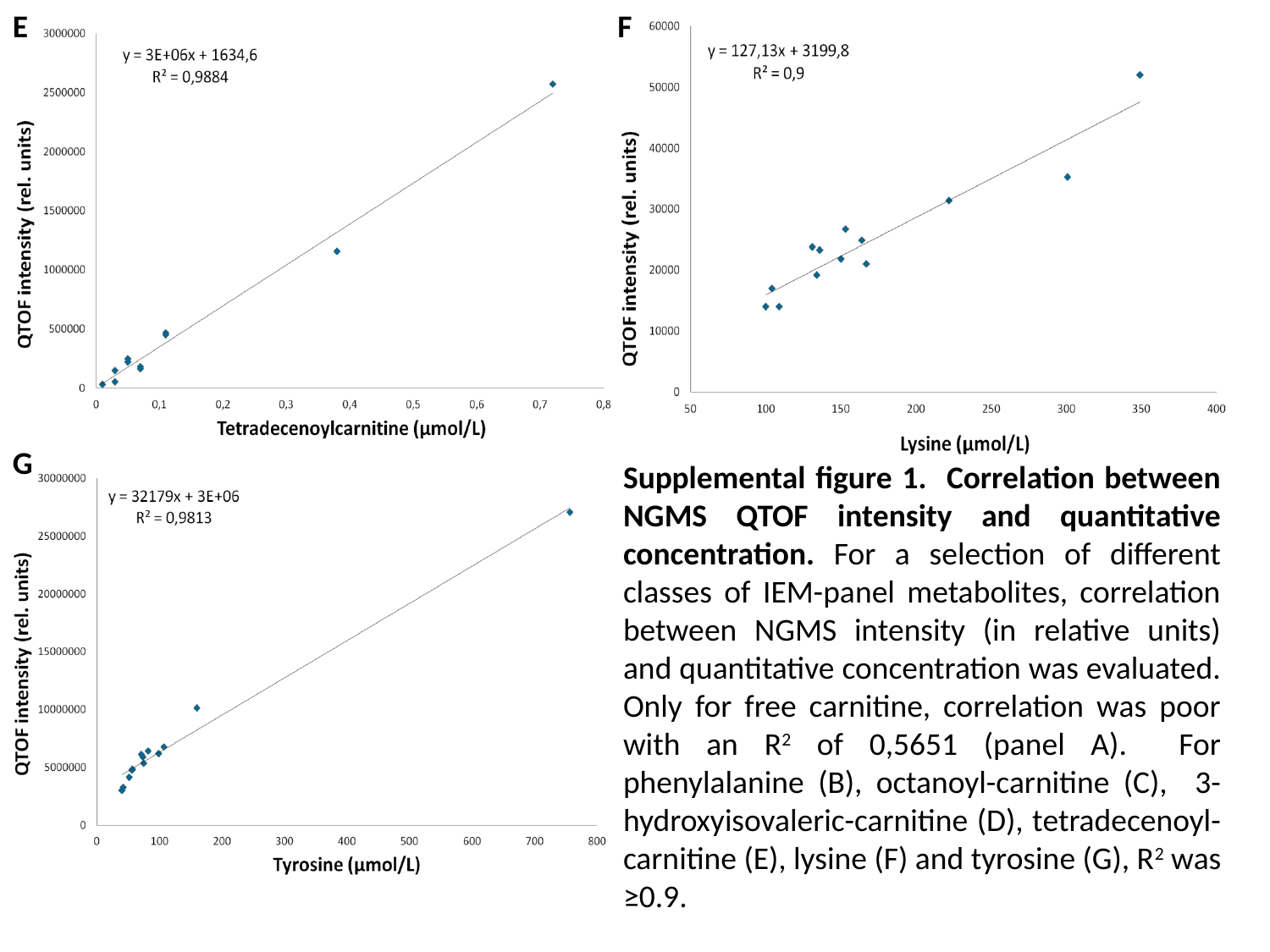

E
F
G
Supplemental figure 1. Correlation between NGMS QTOF intensity and quantitative concentration. For a selection of different classes of IEM-panel metabolites, correlation between NGMS intensity (in relative units) and quantitative concentration was evaluated. Only for free carnitine, correlation was poor with an R2 of 0,5651 (panel A). For phenylalanine (B), octanoyl-carnitine (C), 3-hydroxyisovaleric-carnitine (D), tetradecenoyl-carnitine (E), lysine (F) and tyrosine (G), R2 was ≥0.9.
