# Supplementary material for: Next-generation metabolic screening: targeted and untargeted metabolomics for the diagnosis of inborn errors of metabolism in individual patients
Source: J Inherit Metab Dis. 2018 Feb 16;41(3):337–53. doi: 10.1007/s10545-017-0131-6 (PMC5959972; doi:10.1007/s10545-017-0131-6)
Supplement: Supplementary file 2 — (PPTX 176 kb) [file 10545_2017_131_MOESM2_ESM.pptx]

## Slide 1
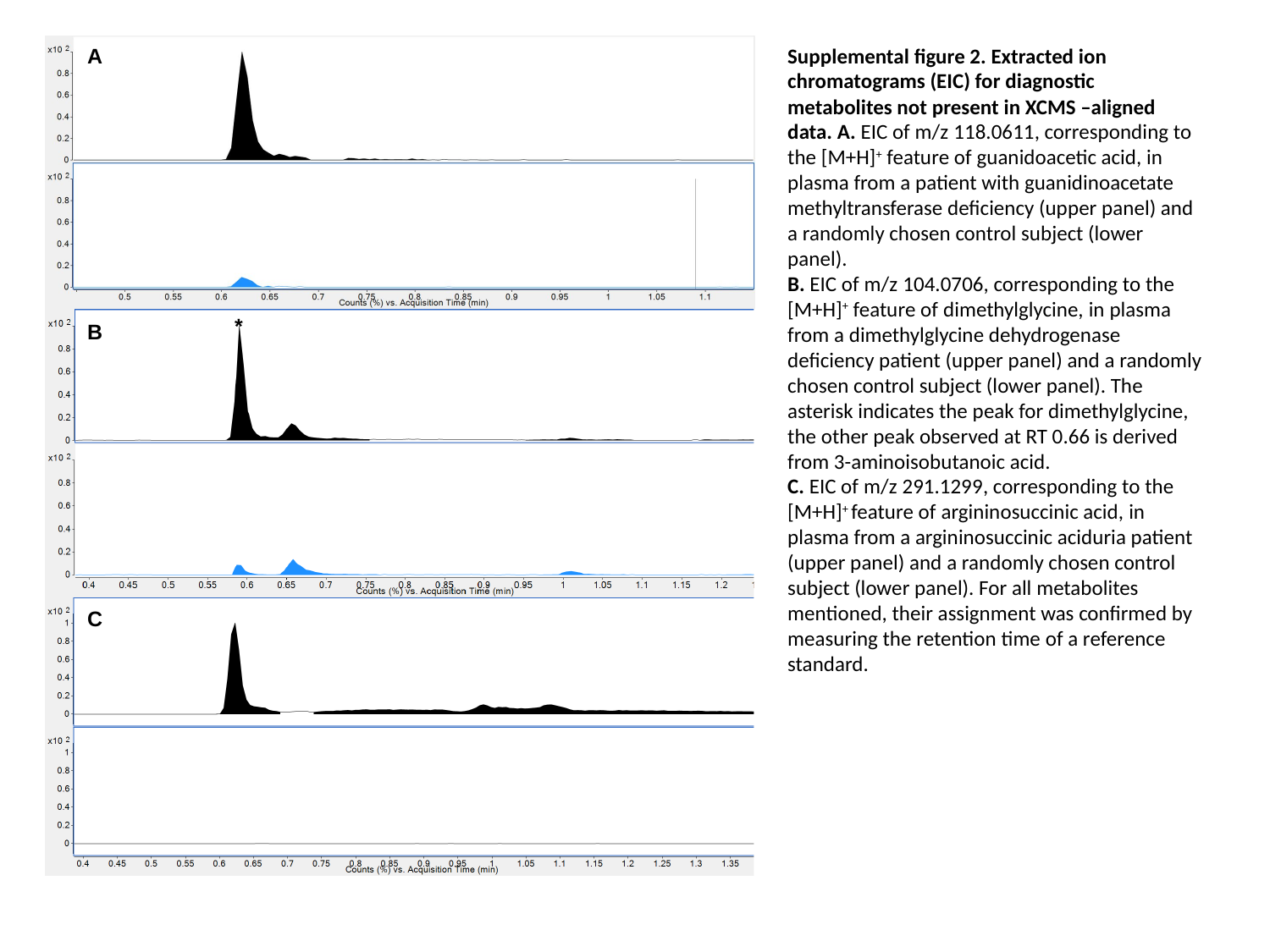

A
Supplemental figure 2. Extracted ion chromatograms (EIC) for diagnostic metabolites not present in XCMS –aligned data. A. EIC of m/z 118.0611, corresponding to the [M+H]+ feature of guanidoacetic acid, in plasma from a patient with guanidinoacetate methyltransferase deficiency (upper panel) and a randomly chosen control subject (lower panel).
B. EIC of m/z 104.0706, corresponding to the [M+H]+ feature of dimethylglycine, in plasma from a dimethylglycine dehydrogenase deficiency patient (upper panel) and a randomly chosen control subject (lower panel). The asterisk indicates the peak for dimethylglycine, the other peak observed at RT 0.66 is derived from 3-aminoisobutanoic acid.
C. EIC of m/z 291.1299, corresponding to the [M+H]+ feature of argininosuccinic acid, in plasma from a argininosuccinic aciduria patient (upper panel) and a randomly chosen control subject (lower panel). For all metabolites mentioned, their assignment was confirmed by measuring the retention time of a reference standard.
*
B
C
