# Supplementary material for: Next-generation metabolic screening: targeted and untargeted metabolomics for the diagnosis of inborn errors of metabolism in individual patients
Source: J Inherit Metab Dis. 2018 Feb 16;41(3):337–53. doi: 10.1007/s10545-017-0131-6 (PMC5959972; doi:10.1007/s10545-017-0131-6)
Supplement: Supplementary file 3 — (PPTX 131 kb) [file 10545_2017_131_MOESM3_ESM.pptx]

## Slide 1
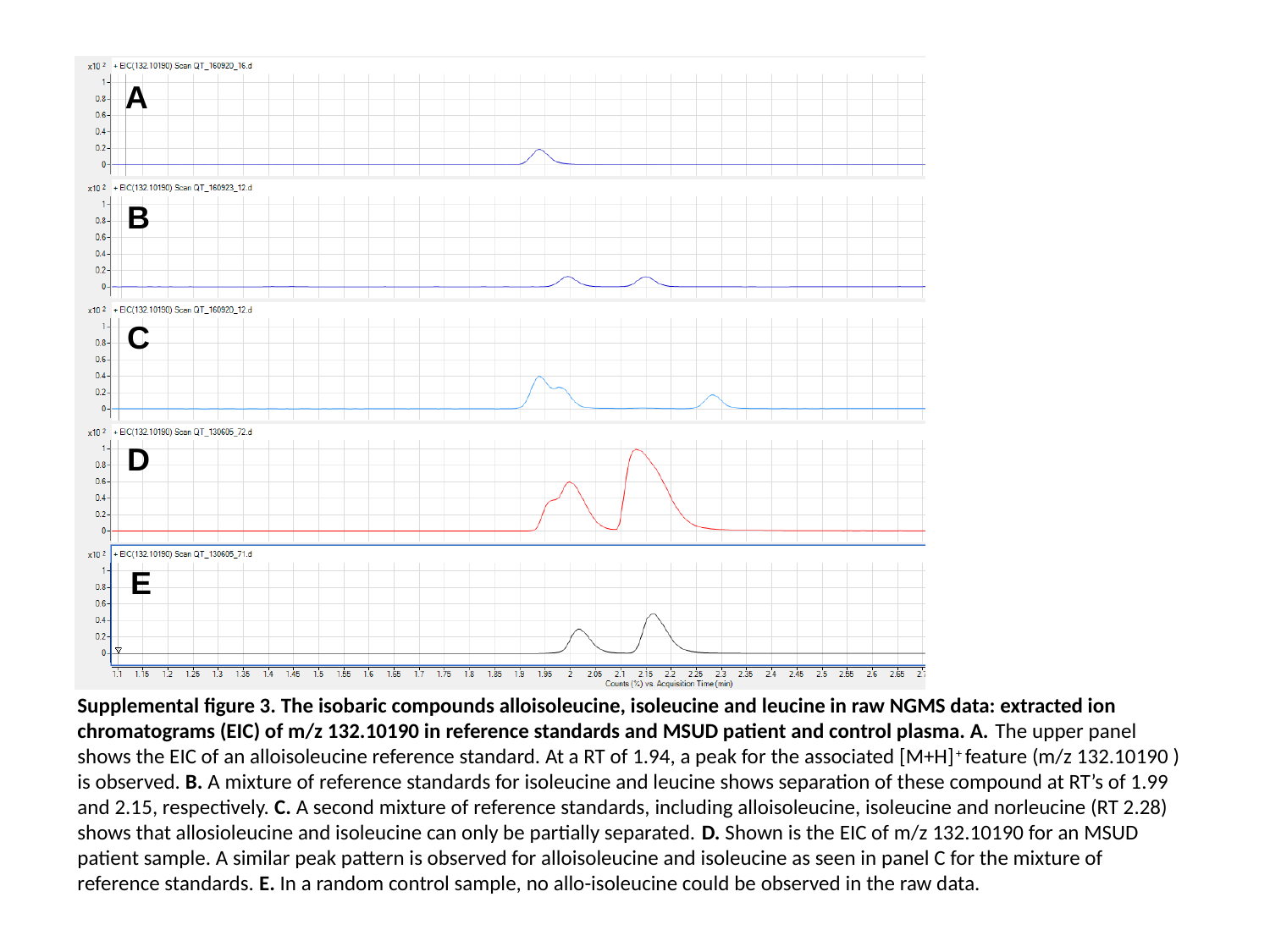

A
B
C
D
E
Supplemental figure 3. The isobaric compounds alloisoleucine, isoleucine and leucine in raw NGMS data: extracted ion chromatograms (EIC) of m/z 132.10190 in reference standards and MSUD patient and control plasma. A. The upper panel shows the EIC of an alloisoleucine reference standard. At a RT of 1.94, a peak for the associated [M+H]+ feature (m/z 132.10190 ) is observed. B. A mixture of reference standards for isoleucine and leucine shows separation of these compound at RT’s of 1.99 and 2.15, respectively. C. A second mixture of reference standards, including alloisoleucine, isoleucine and norleucine (RT 2.28) shows that allosioleucine and isoleucine can only be partially separated. D. Shown is the EIC of m/z 132.10190 for an MSUD patient sample. A similar peak pattern is observed for alloisoleucine and isoleucine as seen in panel C for the mixture of reference standards. E. In a random control sample, no allo-isoleucine could be observed in the raw data.
